# Hypnotism: Its History, Practice, and Theory

**Published:** 1905-03

**Authors:** 


**Hypnotism: its History, Practice, and Theory.** By J. MILNE BRAMWELL, M.B., C.M. Pp. xiv., 478. London: Grant Richards. 1903.

This book can be well recommended to all those interested in hypnotism, whether from the medical or purely scientific standpoint. The subject is treated in an unbiased spirit throughout, and there is no attempt made to force the claims of hypnotism as a curative agent. The work opens with a concise historical survey of the work of the earlier investigators and of the later French schools, with a description of their methods.

There is a very interesting section on the experimental phenomena of hypnotism, in which the post-hypnotic phases receive especial notice; the case of time-appreciation is, with the author's discussion of it, worthy of careful perusal. Dr. Bramwell's views on the therapeutic value of hypnotism in medicine and surgery are given with judicial impartiality. He claims that a good percentage of cures can be obtained in cases of hysteria, obsessions and imperative ideas, and neuralgias. Its use as an anæsthetic is limited; profound anæsthesia is only obtainable in a few of even susceptible patients. The various methods of inducing and terminating hypnosis are reviewed; the author mainly relies upon procuring an expectant attention in the patient and then on suggestion, sometimes using suggestion for the hypnosis and the treatment simultaneously. The use of mechanical aids is not favoured by him. Dr. Bramwell denies that the easy production of hypnosis is any indication of mental feebleness, as has been popularly supposed; rather concentration of mind is requisite in considerable degree. Nor does he admit the likelihood of a post-hypnotic subserviency of subject to operator of a general kind, or in especial any tendency to adopt criminal suggestion. We gather that hypnotism has never been attended with any ill results in the author's practice, and that far from impairing volition it is productive of increased control.

